# Cox-maze III procedure for atrial fibrillation during valve surgery: a single institution experience

**DOI:** 10.1186/s13019-020-01165-4

**Published:** 2020-05-24

**Authors:** Chang-tian Wang, Lei Zhang, Tao Qin, Zhi-long Xi, Lei Sun, Hai-wei Wu, De-min Li

**Affiliations:** Department of Cardiovascular Surgery. Jinling Hospital, Nanjing University, School Medicine, 305 East Zhongshan Road, Nanjing, 210002 PR China

**Keywords:** Maze procedure, Atrial fibrillation, Valve surgery

## Abstract

**Objectives:**

Atrial fibrillation (AF) is the most common sustained cardiac arrhythmia in patients with heart valve disease. Our aim was to summarize our experience and evaluate the efficacy and safety of the Cox maze III procedure combined with valve surgery in patients with AF.

**Methods:**

A retrospective, observational analysis was performed for all consecutive patients underwent maze III procedure combined with valve surgery between October 2015 and June 2019. In this trial, we used a monopolar radiofrequency (RF) ablation in addition to cut and sew technique to treat AF.

**Results:**

66 patients (37 female, 56.1%) with persistent or long-lasting persistent AF associated with valve disease were identified. The mean age was 54.2 ± 8.4 years (range, 30 to 73 years). Overall hospital mortality was 3.0%. The duration of cardiopulmonary bypass and aortic cross clamping was 175.4 ± 32.9 and 115.6 ± 22.8 min respectively. The first 24 h drainage was 488.6 ± 293.3 ml. The postoperative hospital stay was 14.8 ± 8.3 days. The postoperative incidence of permanent pacemaker implantation, reoperation for bleeding, renal failure required hemodialysis, and stroke was 4.5, 1.5, 4.5% and 0 respectively. The frequency of sinus rhythm was 91.7, 93.1, 94.7, 93.3 and 89.5% at 1, 3, 6, 12, and 24 months respectively.

**Conclusions:**

The Cox-Maze III procedure is safe in the surgical treatment of AF associated with valve disease, and efficacious for sinus rhythm maintenance, with low morbidity and mortality.

## Introduction

Atrial fibrillation (AF) is a common cardiac arrhythmia that occurs in 1.5–2.0% of the general population. AF presents in up to 40 to 60% of patients undergoing mitral valve surgery and considerably increases the mortality risk over the years after operation [1]. Despite numerous innovations in contemporary ablation and surgery, their effectiveness and safety have not been rigorously established [2], and the management is variably performed among patients with AF undergoing cardiac surgery. The Cox maze III operation, also called the “cut-and-sew” maze operation, is a complex surgical procedure for the control of atrial fibrillation, and remains the reference standard for the surgical treatment of AF and should still be considered, especially for patients for whom AF ablation is of critical importance [3–5]. But due to surgical complexity and perceived notion of greater morbidity, this operation is not widely adopted among cardiac surgeons [6].

The aim of this study was to evaluate the efficacy and safety of the Cox maze III procedure combined with valve surgery in patients with chronic AF in a single-center retrospective study.

## Materials and methods

This retrospective single-centre observational study was approved by the local ethics review board. We reviewed our valve surgery database to identify patients who underwent surgery combined with Cox maze III procedure for persistent or long-lasting persistent AF between October 2015 and June 2019 at the Department of Cardiovascular Surgery of the Nanjing Jinling Hospital. According to 2014 AHA/ACC/HRS guideline [7], persistent atrial fibrillation was defined as continuous atrial fibrillation for more than 7 days. Long-standing persistent atrial fibrillation was defined as continuous atrial fibrillation for more than 12 months. A total of 66 consecutive patients underwent valve surgery combined with Cox maze III procedure were identified. Approval of the ethics committee of the Jinling hospital was obtained. Data were from each electronic patient’s medical record. The individual consent for the study was waived.

### Surgical management

One cardiac surgeon team performed all operations. All patients were operated through general anesthesia, median sternotomy, using cardiopulmonary bypass with moderate hypothermia (28-30C). The maze operation was performed according to the original description of James Cox [8]. We used monopolar RF ablation in addition to cut and sew technique to treat AF. After full cardiopulmonary bypass is established, the right atrial maze is performed, including the excision of right atrial appendage, a lateral incision from the base of the excised atrial appendage toward the inferior vena cava (IVC), a posterior longitudinal incision from well into the SVC to well into the IVC, a T incision from posterior longitudinal incision to the tricuspid valve annulus above the IVC cannula, and the anterior right atrial counter incision from anteromedial border of the excised right atrial appendage to the anteromedial tricuspid valve annulus. Monopolar RF ablation using either the AtriCure device (AtriCure, Inc., Cincinnati, Ohio) or the Cardioblate device (Medtronic, Inc., Minneapolis, Minn) is applied at the tricuspid end of the T incision and the anteromedial incision. Then the aorta is clamped and myocardial protection is achieved by antegrade cold HTK solution. The left atrial appendage is amputated and closed at its base, suture reinforced with felt. A left atriotomy is performed in the interatrial groove, and atrial septum is divided and terminated at the bottom of the fossa ovalis. The left atriotomy is extended inferiorly across the posterior left atrial free wall between the mitral valve and the orifices of the inferior pulmonary veins. Likewise, the superior portion of the left atriotomy is extended around the lip of the left superior pulmonary vein orifice. The small bridge of tissue between the appendage amputation site and the two ends of the pulmonary vein is left intact and applied with RF ablation. The final posterior vertical incision extends from the right inferior pulmonary vein to the mitral valve annulus is made. The coronary sinus and the end of this incision adjacent to the mitral valve annulus is subjected to RF ablation. Valve surgery is performed. The right atrial incisions are closed after aortic cross-clamp released. All atrial incisions are closed with double running full-layer mattress sutures.

### Data collection

Patients’ demographics and clinical data were recorded. Follow-up information was obtained from subsequent clinic visits. The primary safety endpoints were the rates of hospital death and major adverse events postoperatively (i.e., permanent pacemaker implantation, reoperation for bleeding, renal failure required hemodialysis, and stroke). The secondary endpoint was the restoration of sinus rhythm (SR). The heart rhythm was evaluated mainly on the basis of 12-lead electrocardiogram (ECG) and partly by 24-h Holter ECG obtained at 1, 3, 6, 12, and 24 months after surgery.

### Statistical analysis

All statistical analyses were performed with the SPSS Statistics, version 21 software (SPSS Inc., Armonk, NY). Standard definitions were used for patient variables and outcomes. Continuous variables are expressed as mean ± SD and categorical variables are expressed as percentages.

## Results

Between October 2015 and June 2019, a total of 66 patients underwent cut and sew Cox maze III procedure concomitant valve surgery (Table 1). Mean age was 54.2 ± 8.4 years (range, 30-73 years) and 37 (56.1%) were females. Preoperatively, 22 patients (33.3%) were in New York Heart Association (NYHA) functional class III or IV. The mean left ventricular ejection fraction (LVEF) was 55.7 ± 9.3%. The mean left atrial diameter (LAD) and mean left ventricular diameter (LVD) was 53.1 ± 7.3 mm and 51.7 ± 8.0 mm respectively. Preoperative patients’ demographic data are summarized in Table 1.

**Table 1:** Preoperative clinical characteristics

Characteristic	Cox III plus valve surgery (*n* = 66)
Female sex - no. (%)	37 (56.1)
Age - yr	54.2 ± 8.4
Hypertension - no. (%)	10 (15.1)
Diabetes mellitus - no. (%)	5 (7.6)
History of stroke - no. (%)	4 (6.0)
NYHA class III or IV - no. (%)	22 (33.3)
Left ventricular ejection fraction - %	55.7 ± 9.3
Left atrial diameter - mm	53.1 ± 7.3
Left ventricular diameter - mm	51.7 ± 8.0

Notes: Data are presented as numbers (%) or as mean ± SD. *NYHA*, New York Heart Association

The concomitant valve operations performed during Cox maze III procedure included mitral valve replacement (MVR) in 25 patients (37.9%), MVR plus tricuspid valve repair (TVr) in 13 (19.7%), MVR plus aortic valve replacement (AVR) in 9 (13.6%), MVR plus AVR plus TVr in 5 (7.6%), MVR plus TVr plus CABG in 1 (1.5%), MVr in 3 (4.5%), MVr plus TVr in 6 (9.1%), MVr plus AVR in 2 (3.0%), and AVR in 2 (3.0%). The LA appendage was amputated in all patients. The median cardiopulmonary bypass and crossclamp time was 175.4 ± 32.9 min (range, 111–248 min) and 115.6 ± 22.8 min (range, 78–179 min) respectively (Table 2).

**Table 2:** Operative Characteristics of the Patients

Operative variable	Cox III plus valve surgery (*n* = 66)
Combined valve surgery - no.	
MVR - no. (%)	25 (37.9)
MVR plus TVr - no. (%)	13 (19.7)
MVR plus AVR - no. (%)	9 (13.6)
MVR plus AVR plus TVr - no. (%)	5 (7.6)
MVR plus TVr plus CABG - no. (%)	1 (1.5)
MVr - no. (%)	3 (4.5)
MVr plus TVr - no. (%)	6 (9.0)
MVr plus AVR - no. (%)	2 (3.0)
AVR - no. (%)	2 (3.0)
Duration of cardiopulmonary bypass - min	175.4 ± 32.9
Duration of aortic cross-clamping - min	115.6 ± 22.8

Notes: Data are presented as numbers (%) or as mean ± SD. *MVR* valve replacement, *MVr* Mitral valve repair, *AVR* Aortic valve replacement, *TVr* Tricuspid valve repair, *CABG* Coronary artery bypass grafting

The overall hospital mortality was 3% (*n* = 2). One patient died 28 days after operation from sepsis and multi-organ failure. The second patient underwent re-do MVR plus Cox maze III had normal postoperative course, but died on postoperative day 9 from sudden cardiac arrest after being in normal sinus rhythm for few days. The incidence of 30 days postoperative atrial arrhythmia (POAA) was 9.1% (*n* = 6), and the stroke, re-exploration for bleeding, renal failure requiring hemodialysis, superficial wound infection (SWI), tracheostomy was 0 (*n* = 0), 1.5% (*n* = 1), 4.5% (*n* = 3), 4.5% (*n* = 3), tracheostomy 1.5% (*n* = 1) respectively. 3 patients (4.5%) required the permanent pacemaker implantation (PPI) postoperatively, in which, 2 patients underwent MVR plus TVr (15%), and 1 patient underwent MVR plus AVR plus TVr (20%). The mean volume of the first 24-h drainage was 488.6 ± 293.3 ml (range, 155-1200 ml). One patient suffered from continuous chest tube output of blood greater than 1000 ml postoperative 6 h. He was transferred to the operation room again to stop bleeding. The bleeding was due to coagulopathy, not surgical bleeding. In this group, the mean total length of postoperative hospital stay was 14.8 ± 8.3 days (range, 7-51 days)(Table 3).

**Table 3:** Postoperative Clinical Outcomes

Variable	Cox III plus valve surgery (*n* = 66)
Hospital mortality(30 days)- no. (%)	2 (3.0)
Drainage (24hs) -ml	488.6 ± 293.3
Postoperative hospital stay-d	14.8 ± 8.3
POAA (30 days) - no. (%)	6 (9.1)
Stroke	0
Reoperation for bleeding - no. (%)	1 (1.5)
Renal failure (hemodialysis) - no. (%)	3 (4.5)
SWI - no. (%)	3 (4.5)
Tracheostomy - no. (%)	1 (1.5)
Postoperative permanent pacemaker - no. (%)	3 (4.5)

Notes: Data are presented as numbers (%) or as mean ± SD. *POAA* Postoperative atrial arrhythmia, *SWI* Superficial wound infection

**Fig. 1:**
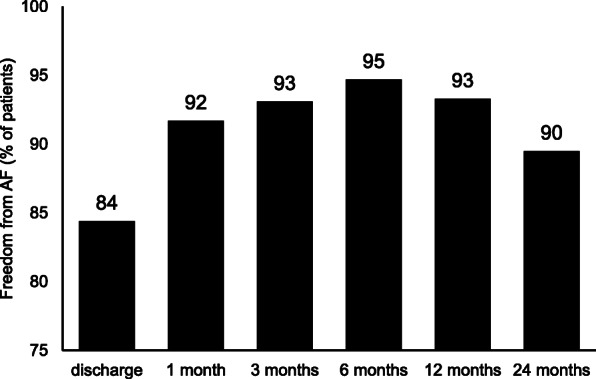
Freedom from Atrial Fibrillation

Clinical follow-up was performed for 1–24 months postoperatively. 12-lead ECG or 24-h Holter ECG were taken during each follow-up visit in all survivor patients (64 patients) at hospital discharge, in 60 (93.8%) at 1 month follow-up, in 58 (90.6%) at 3 months, in 57 (89.1%) at 6 months, in 45 (70.3%) at 12 months, and in 38 (59.4%) at 24 months. Freedom from AF without anti-arrhythmia drugs (AADs) at the last follow-up point was 84.4, 91.7, 93.1, 94.7, 93.3, 89.5%, respectively (Fig. 1). At 3 months postoperatively, 2 of 3 patients received the PPI postoperatively converted the sinus rhythm. One patient with junctional rhythm at discharge required PPI at the third month after surgery. The outcomes of heart rhythm follow-up are listed in Table 4.

**Table 4:** The heart rhythm follow-up data

Time	follow-up cases	follow-up rate(%)	Sinus rhythm	Atrial arrhythmia	Junctional rhythm	Frequency of SR (%)
Discharge	64	100	54	5	5	84.4
1 month	60	93.8	55	3	2	91.7
3 month	58	90.6	54	2	2	93.1
6 month	57	89.1	54	2	1	94.7
12 month	45	70.3	42	2	1	93.3
24 month	38	59.4	34	3	1	89.5

Notes: Data are presented as numbers (%) or as mean ± SD. *SR* Sinus rhythm

## Discussion

The Cox maze III operation (sometimes called the “cut-and-sew” maze operation) is a complex surgical procedure for the control of AF, and is extensive and time consuming, requires great surgical skill. Our initial concern was unsafe, especially the potentially dangerous for bleeding, to perform the maze III procedure concomitant valve surgery because of the significantly increased cardiopulmonary bypass and aortic cross-clamp time. In this study, we described the experience of 66 patients with persistent or long-standing persistent AF who underwent maze III procedure combined with valve surgery over a 3-year period. The population had a median age of 54 years. The in-hospital mortality was 3%, suggesting that the Cox maze III procedure combined with valve surgery is both feasible and safe in a carefully selected population. Our in-hospital mortality was in keeping with previous reports describing the operation of maze III procedure combined with valve surgery [3, 5, 9–11]. Despite the increased duration of CPB and aortic cross-clamp time, the incidence of major postoperative complications, including bleeding, renal failure, stroke and SWI, were low in this study. The results could be attributed to the fact that the improvement of modern intro-operative myocardial protection strategy. In this population, no one was complicated by heart failure, required IABP or ECMO support. However, the cohort in this study was low risk (mean age 54.2 years, mean LVEF 55.7%), which might have influenced the results. So we should be cautious when considering the possibility of maze III procedure plus valve surgery in those high risk patients, such as elderly, lower LVEF.

In this trial, we found that the rate of freedom from atrial fibrillation in 24 months after Cox maze III procedure was over 90%. In recent years, new technologies and approaches to surgical AF ablation have been evolved to simplified lesion sets and shorten the time, but the issue of ensuring completely transmural lesions remains unresolved. In a randomized multicenter trial involving patients with persistent or long-standing persistent AF who were undergoing mitral-valve surgery, Gillinov AM et al. [2] reported the freedom from AF in the first year after surgery was 63.2% in the ablation procedures (pulmonary-vein isolation or biatrial maze procedure) group. The “cut-and-sew” maze III operation can assure the transmurality and eliminates this concern. Stulak et al. [3] reported the patients undergoing the Cox maze III procedure concurrent with isolated mitral valve surgery resulted in significantly greater freedom from AF without antiarrhythmic medication compared with any other procedure for AF ablation within 1 year postoperatively (87% vs 70%, *P* = 0.04) and after 5 years postoperatively (75% vs 52%, *P* = 0.03). Many of the studies have shown for both catheter and surgical ablation that left atrial enlargement is a predictor of failure [12]. The study reported by Ishii Y [13] revealed that preoperative LAD of ≧58.0 mm was a significant risk factor for an AF recurrence after full maze procedure, including cut and sew procedure and device ablation. In their report, the AF cure rates were 85, 59, and 42% at 1, 5, and 10 years in the population who had a LAD = 64.2 mm (58.0–82.0 mm). However, in the population who had a LAD = 54.2 (52.0–57.0 mm), the AF cure rates were 99, 85, and 68% at 1, 5, and 10 years. It is interesting that the data of this population was similar to this trial, so we compared them as shown in Table 5. In the present trial, the mean diameter of preoperative left atrial was 53 mm, and the mean LVEF was 55.7%. These factors might have improved the likelihood of ablation success in our study. Meanwhile, the LAD ranged from 40 to 70 mm, and about 30% patients’s LAD were larger than 58.0 mm in this trial, which might contribute to the results that AF cure rates are just between the above two groups.

**Table 5:** The comparison of clinical characteristics between this trial and groups from literature

Characteristic	This trial (*n* = 66)	Group 3 from Ishii Y[13] (*n* = 66)	Group 4 from Ishii Y[13] (*n* = 62)
Female - no. (%)	37 (56.1)	25 (37.9)	19 (30.6)
Age - yr	54.2 ± 8.4	63.2 ± 13.5	64. ± 10.5
NYHA class III or IV - no. (%)	22 (33.3)	11 (16.7)	10 (16.1)
LVEF - %	55.7 ± 9.3	60.3 ± 12.6	62.34 ± 10.6
CPB time (min)	175.4 ± 32.9	219 ± 43	229 ± 62
Aortic cross-clamping time (min)	115.6 ± 22.8	160 ± 38	182 ± 48
Left atrial diameter - mm	53.1 ± 7.3 (40–70)	54.2 ± 1.6(52–57)	64.2 ± 5.6 (58–82)
Frequency of SR 1 year (%)	93.3	99	85
Frequency of SR 2 year (%)	89.5	85 (5 year)	59 (5 year)

Notes: Data are presented as numbers (%) or as mean ± SD. CPB Cardiopulmonary bypass, LAD Left atrial diameter, LVEF Left ventricular ejection fraction, NYHA New York Heart Association, SR Sinus rhythm

The need for PPI after operation remains a matter of concern for the Cox maze III procedure. In our study, 2 of 3 patients received PPI before discharge converted sinus rhythm at 3 months postoperatively, meanwhile one patient with junctional rhythm at discharge required PPI. The incidence of PPI in two and three associated valve procedures was 15% vs 20% respectively. The results related to the need for PPI in the literatures were different. In a case-matched study [14], Stulak et al. found that new PPI was required in significantly more patients in the radiofrequency group than in the cut and sew group (25% versus 5%). In a single-center cohort of Cox maze III procedure concomitant cardiac surgery, Fernando A et al. [11] found that the PPI was required in 3.6% of total, and 18.2% of those with three associated valve procedures. A cumulative meta-analysis of randomized controlled trials (RCT) on clinical outcomes of surgical ablation versus no ablative treatment in all patients with cardiac surgery demonstrated that there were no significant differences between surgical ablation versus no ablation in terms of pacemaker implantations [15]. One reason that new permanent pacemakers are required following a maze procedure is premature pacemaker implantation for a temporary junctional rhythm immediately postoperatively [16]. In the present study, the rate of junctional rhythm in the immediate postoperative period following maze procedure was 7.9% (5/64) overall.

The following factors limited this study. First, it was a retrospective trial and possessed all the inherent limitations of this type of study design. Second, the rhythm was evaluated mainly on the basis of 12-lead ECG and partly by 24-h Holter ECG, which might tend to overestimate the clinical success, compared with long-term monitoring. The HRS guidelines (2012) recommend that a 1- to 7-day Holter monitoring is an effective way to identify frequent asymptomatic recurrences of AF. We have begun to follow patients with 7-day Holter monitoring during follow-up in the current era. Finally, our follow-up data were not available for all patients, and had a small sample size. A larger sample and longer follow-up are required for a future study. This is just the beginning of our study and will continue to do better.

## Conclusions

In this retrospective study involving patients with persistent or long-standing persistent atrial fibrillation, the combination of the Cox maze III procedure at the time of valve surgery is safe and effective with low morbidity and mortality, offers great freedom from AF at the early stage postoperatively.

## Data Availability

All data generated or analysed during this study are included in this published article.

## Abbreviations

AADs: Anti-arrhythmia drugs
AF: Atrial fibrillation
AVR: Aortic valve replacement
CABG: Coronary artery bypass grafting
CPB: Cardiopulmonary bypass
ECG: Electrocardiogram
ECMO: Extracorporeal membrane oxygenation
IABP: Intra-aortic balloon pump
IVC: Inferior vena cava
LAD: Left atrial diameter.
LVD: Left ventricular diameter
LVEF: Left ventricular ejection fraction
MVR: Valve replacement
MVr: Mitral valve repair.
NYHA: New York Heart Association
POAA: Postoperative atrial arrhythmia
PPI: Permanent pacemaker implantation
SR: Sinus rhythm
SVC: Superior vena cava
SWI: Superficial wound infection
TVr: Tricuspid valve repair
